# Assessment of the Effects of Corporate Governance on the Competitiveness of Pharmaceutical Companies Through Improved Total Factor Productivity

**DOI:** 10.5812/ijpr-169077

**Published:** 2026-09-23

**Authors:** Mohammad Hossein Motevalli, Farzad Peiravian, Mohammad Peikanpour, Mohammad Moien Mansouri, Seyyed Ahmad Askari

**Affiliations:** 1 Department of Pharmacoeconomics and Pharma Management, School of Pharmacy, Shahid Beheshti University of Medical Sciences Tehran Iran; 2 Cell Therapy and Hematopoietic Stem Cell Transplantation Research Center, Research Institute for Oncology, Hematology and Cell Therapy Tehran Iran; 3 Department of Traditional Pharmacy, Traditional Medicine and Materia Medica Research Center, School of Traditional Medicine, Shahid Beheshti University of Medical Sciences Tehran Iran

**Keywords:** Competitive Advantage, Organizational Policy, Pharmaceutical Industry, Iranian Pharmaceutical Companies, Ergonomics, Work Schedule, Cognitive Function, Alertness, Mental Workload

## Abstract

**Background:**

Corporate governance, productivity, and competitiveness are widely recognized as key determinants of firm performance and long-term sustainability. Although each construct has been extensively studied individually, their interrelationships, particularly within the pharmaceutical industry of developing economies, remain insufficiently understood. This gap is especially relevant given the strategic and economic importance of the pharmaceutical sector.

**Objectives:**

This study aimed to assess the relationships among corporate governance, competitiveness, and productivity in Iranian pharmaceutical companies and to develop a model that better explains the interactions among these determinants.

**Methods:**

This cross-sectional study was conducted in three phases among Iranian pharmaceutical manufacturing companies. First, context-specific, validated questionnaires were developed to assess corporate governance, productivity, and competitiveness. Second, these instruments were administered to senior executives (Chief Executive Officers [CEOs], board members, Chief Financial Officers [CFOs], and Chief Commercial Officers [CCOs]) at 44 randomly selected companies. Finally, statistical analyses, including Pearson and partial correlations, independent t-tests, and bootstrapped mediation analysis, were performed to evaluate the relationships among the study variables.

**Results:**

A total of 151 completed questionnaires were analyzed (response rate: 85.8%). Productivity showed a strong positive correlation with competitiveness (r = 0.780, P < 0.001) and a moderate positive correlation with corporate governance (r = 0.419, P = 0.005). By contrast, no significant direct correlation was observed between corporate governance and competitiveness. Mediation analysis indicated that productivity mediated the relationship between corporate governance and competitiveness, indicating a substantial indirect effect. In addition, companies producing biological medicines had significantly higher productivity and competitiveness scores.

**Conclusions:**

The findings suggest a partially interdependent model in which corporate governance enhances competitiveness primarily through its positive effect on productivity rather than through a direct pathway. Strengthening governance structures to improve decision-making and resource utilization may therefore be an effective strategy for increasing productivity and, ultimately, competitiveness in the pharmaceutical industry.

## 1. Background

The modern economic landscape is characterized by intense competition, in which the long-term success and sustainability of enterprises are closely tied to their competitive capabilities (1). Firm-level competitiveness is generally defined as a company’s ability to produce higher-quality goods and services at lower costs than its domestic and international rivals, or to possess other distinct competitive advantages that attract customers (2). Such competitiveness is fundamental to increasing long-term profitability and enhancing a company’s ability to compensate employees and deliver superior returns to its owners (3, 4). Firm-level competitiveness is typically associated with market performance and productivity (5, 6). Although competitiveness is also defined at the national level as a country’s ability to create, produce, distribute, or service products in international trade while simultaneously increasing the return on its resources (2), this study emphasizes firm-level competitiveness because of its cumulative positive effects on a society’s economy, fostering economic growth and improving public welfare.

Beyond competitiveness, productivity and corporate governance are pivotal concepts for strong industrial performance. Productivity is generally defined as the ratio of the total value of products generated to the total value of all inputs consumed. It comprises two core components: efficiency and effectiveness. Efficiency, as the primary aspect, refers to the ratio of output obtained to a given set of inputs. Efficacy, in contrast, relates to achieving the desired goals or results set by the producer (7, 8). Although maximizing efficiency is necessary to achieve maximum productivity, a sole focus on efficiency without also maximizing effectiveness is insufficient (9). Productivity is crucial for maintaining competitiveness, at both the organizational and national levels, and for ensuring sustainable economic development (10-12). Its importance is further underscored by its vulnerability to various external and internal factors. Financial crises, often exacerbated by sanctions and currency fluctuations in contexts such as Iran, introduce uncertainty in prices and interest rates, disrupt pricing systems, stifle investment, and lead to suboptimal resource allocation and a decline in productivity (13). Although several distinct measures of productivity exist, Total Factor Productivity (TFP) is a broader and more comprehensive measure than single-factor indicators such as labor productivity or financial ratios such as return on assets (14, 15). Whereas labor productivity captures output relative to a single input and financial indicators reflect performance outcomes, TFP reflects the efficiency with which firms transform multiple inputs (including capital, labor, technology, and organizational capabilities) into output (15, 16). In knowledge-intensive industries such as pharmaceuticals, in which innovation processes, regulatory compliance, human capital, and technological integration jointly shape performance, TFP provides a more appropriate conceptual representation of firm-level productivity (17). In the present study, productivity is treated as a multidimensional construct intended to approximate the underlying concept of TFP by capturing efficiency and effectiveness across financial, human resource, process, and material domains. Conceptually, TFP functions as an intermediary mechanism linking corporate governance to competitiveness: governance structures influence decision quality, resource allocation, and managerial accountability, which in turn enhance productive efficiency and ultimately strengthen competitive positioning.

Furthermore, corporate governance is a crucial characteristic for a company’s long-term survival, sustained growth, and enhanced productivity. Corporate governance is a broad term encompassing the methods, structures, and processes through which a company’s operations are managed and directed (18). Its primary objective is to mitigate incentive problems arising from the separation of ownership and control in firms in which managers differ from business owners (19). According to the Organisation for Economic Co-operation and Development (OECD), sound corporate governance fosters a climate of trust, transparency, and accountability that is essential for promoting long-term investment, financial stability, and business integrity, thereby supporting stronger and more inclusive societal growth (20). Key elements of corporate governance include ownership structure, capital structure, investor relations with the management team, and board structure. Understanding corporate governance is beneficial because the costs associated with divergent views between owners and managers can be substantial and may lead to reduced company productivity (21-23). Recurrent global financial crises have highlighted the importance of robust corporate governance and effective managerial efforts to improve firm productivity.

Numerous factors are recognized to be associated with each of these three aspects. In addition to external factors affecting these aspects, understanding their interrelationships can facilitate the identification of causal links and bottlenecks on which strategic decision-making can be based. Appropriate implementation of corporate governance structures is associated with higher competitiveness (24). Moreover, competitiveness can lead to higher effectiveness, which in turn can result in increased productivity. However, despite these findings, it remains unclear how these aspects are related to one another or whether a correlational relationship exists among them (5).

Among the many industries in which this intricate web of organizational performance metrics can be measured, the pharmaceutical industry is a critical global sector, contributing substantially to public health and national economies. Its high value-added nature positions it as a wealth generator for any country. Medicinal products, beyond being a strategic health concern, profoundly affect economic advancement. Despite the well-recognized importance of productivity in organizational value creation, extensive scientific and academic research in this area remains limited, particularly within the economic ecosystems of developing countries, underscoring the need for studies in this domain.

Despite the extensive literature examining corporate governance, productivity, and competitiveness separately, limited empirical work has explored their interrelationships within a unified framework, particularly in developing economies and within the pharmaceutical industry. Existing studies typically rely on financial indicators or single-dimension productivity measures and rarely investigate productivity as a potential mediating mechanism linking governance structures to firm-level competitiveness. Furthermore, few studies have attempted to operationalize a multidimensional representation of productivity tailored to the structural characteristics of the pharmaceutical sector. This study addresses these gaps by empirically examining the association among corporate governance, productivity conceptualized as a proxy for TFP, and competitiveness within Iranian pharmaceutical companies, and by proposing a context-specific measurement framework for capturing productivity across multiple organizational domains.

## 2. Objectives

This study examined the interplay among corporate governance, productivity, and competitiveness in the Iranian pharmaceutical industry. It aimed to assess the associations among these constructs and to develop an industry-specific measure of productivity by identifying key productivity indicators. The findings were intended to inform strategies to improve the performance and long-term sustainability of pharmaceutical companies.

To achieve these objectives and provide a comprehensive understanding, the study was conducted in three distinct phases. The first phase focused on the qualitative development of context-specific indicators and questionnaires relevant to the concepts under investigation in the pharmaceutical industry ecosystem. The second phase used these instruments to empirically measure the dimensions of corporate governance, productivity, and competitiveness in Iranian pharmaceutical companies. The third phase integrated the findings from the preceding phases to propose a refined model, identifying specific dimensions and indicators that require strengthening to enhance the competitiveness of the Iranian pharmaceutical industry in the global pharmaceutical system. This phased approach ensured the development of locally relevant measurement tools and a holistic assessment of the complex relationships involved.

## 3. Methods

This cross-sectional study was conducted in three phases. It aimed to assess the relationships among corporate governance, competitiveness, and productivity in Iranian pharmaceutical companies and to develop a model that better explains the interactions among the determinants of these aspects. To achieve this goal, the status of corporate governance, competitiveness, and productivity needed to be measured reliably in a sample of pharmaceutical companies. Accordingly, the first phase involved developing appropriate instruments to measure these metrics. In the second phase, these instruments were used to measure the target metrics. Finally, statistical analyses were performed to evaluate the dependence of the metrics on one another.

### 3.1. Variables and Measurement Instruments

The population of interest comprised Iranian pharmaceutical companies, and each company was considered an observation. Given the main aim of this article, the primary variables were corporate governance, competitiveness, and productivity. Due to the inherent properties of these variables, including their subjectivity and abstract nature, the most accurate and unbiased available instrument for assessing them was a questionnaire. Additional binary secondary variables, including stock availability status, privatization status, being a subsidiary of a holding company, high-tech medicine production, biological medicine synthesis, a history of activity in international markets as an exporter, and having a distribution division, were also measured for each observation.

### 3.2. Questionnaires and Their Properties

Given the nature of corporate governance, competitiveness, and productivity, a well-designed questionnaire can reduce subjectivity and improve accuracy. First, the structure and domains of each questionnaire were determined. In this study, the validity and reliability of each questionnaire were assessed to ensure the quality of the extracted data. Validity was evaluated by an expert panel based on comprehensiveness, clarity, relevance, and necessity. Experts assessed each item according to these content-validity criteria, and the Content Validity Index (CVI) and Content Validity Ratio (CVR) were subsequently calculated. Experts were eligible if they 1) were active in a pharmaceutical-industry field, including management, academia, regulation, or commerce, and 2) had at least 5 years of work experience. These experts used an agreed-upon framework of comprehensiveness, clarity, relevance, and necessity for the validation assessment. The CVR values ranged from 0.57 to 1.00. Nine items with CVR values below the acceptable threshold of 0.62 were excluded. The expert-panel assessment demonstrated satisfactory content validity, with CVI values ranging from 0.78 to 1.00. To reduce common method variance, questionnaires were distributed across different executive roles within each company, and respondents were instructed to complete only the sections aligned with their areas of expertise. After 30 observations had been completed for each questionnaire, reliability was assessed using Cronbach’s alpha, as reported in Table 1. Cronbach’s alpha values between 0.7 and 1 are generally considered acceptable (25).

**Table 1.** Questionnaires and Their Reliability Status

Questionnaire	Number of Questions	Cronbach’s Alpha
** Corporate governance **	101	0.925
**Competitiveness**	46	0.904
**Productivity**	79	0.935

#### 3.2.1. Corporate Governance

A questionnaire comprising 101 questions was developed based on the framework presented in the International Finance Corporation’s (IFC) 2014 corporate governance scorecard and the OECD Principles of Corporate Governance 2004 (20). The questions were categorized into five categories: 1) shareholders' rights; 2) minority shareholder protection and equitable treatment of shareholders; 3) stakeholder involvement in governance; 4) transparency and disclosure; and 5) the role of the board of directors. A group of experts, selected based on their expertise in corporate governance, assessed the validity of the proposed questionnaire. The Cronbach’s alpha coefficient of 0.925 was well within the suggested range, indicating high internal consistency.

#### 3.2.2. Competitiveness

A questionnaire previously developed by Shabaninejad et al. was used to assess competitiveness (26). This questionnaire contains 46 questions. Although the construct validity and reliability of this questionnaire have already been demonstrated by Shabaninejad et al., both validity and reliability were reassessed. As with the corporate governance questionnaire, the expert panel assessed validity. Cronbach’s alpha was 0.904, indicating a high level of internal consistency.

#### 3.2.3. Productivity

Unlike the two previous questionnaires, which were pre-developed instruments for measuring their corresponding metrics, the literature review for the productivity questionnaire indicated a lack of a valid and reliable metric. Therefore, a questionnaire specific to the Iranian pharmaceutical market was designed and developed. To this end, several steps were undertaken. First, following a thorough literature review, a list of core concepts relevant to productivity was compiled, and a framework for structuring interviews was constructed. A group of experts was selected to conduct semi-structured interviews to identify the key metrics that the questionnaire should measure. These experts were provided with an interview guide in advance. The guide was developed based on a previously established framework. The interview transcripts underwent thematic analysis to extract a thematic network and to prioritize core concepts based on their frequency of mention. Thematic analyses seek to uncover salient themes in a text at different levels, and thematic networks facilitate the structuring and depiction of these themes (27). Next, a comparative analysis was conducted to reconcile the concepts derived from the literature review with the themes identified through thematic analysis. These steps yielded a list of 130 basic themes. This list was then presented to two experts for further refinement. Fifty-one themes were omitted due to similarity and overlap with other themes, as well as the risk of bias from multicollinearity. The resulting network comprised 79 basic themes and four organizing themes, on which the questionnaire was developed.

The questionnaire consisted of 79 questions, divided into four facets and two domains. The four facets were 1) financial metrics, 2) human resource metrics, 3) resource and material metrics, and 4) process metrics. The two domains were efficiency and efficacy. Similar to the other two questionnaires, overall validity was assessed by an expert panel. Cronbach’s alpha was calculated for each facet individually and for the overall questionnaire.

Although productivity is traditionally quantified using objective input–output data, firm-level financial and operational data were not uniformly accessible across companies. Therefore, a multidimensional questionnaire was developed to capture organizational determinants theoretically underlying Total Factor Productivity, including the efficiency of resource utilization, process optimization, human capital performance, and financial management practices. The instrument was designed to reflect structural and operational components that contribute to TFP rather than perceived productivity alone.

### 3.3. Data Collection and Sampling

Sample size calculation using a priori power analysis for Pearson’s correlation coefficients, assuming a moderate correlation (expected Pearson correlation coefficient = 0.4) and a two-sided alternative hypothesis, suggested that 44 observations should be included. In addition, a study with a similar focus in the more diverse ecosystem of Chinese pharmaceutical companies used 47 observations to assess 216 companies (28). At the time of data collection, the population of pharmaceutical synthesizing companies comprised 144 companies. Cluster random sampling was used to increase feasibility, and 44 companies were enrolled. In each company, the Chief Executive Officer (CEO), one board member, the Chief Financial Officer (CFO), and the Chief Commercial Officer (CCO) were each provided with the three questionnaires but were instructed to complete only the parts most relevant to their areas of expertise. Overall, 176 questionnaires were sent to 44 companies between September 2023 and May 2024. Twenty-five questionnaires were not returned, and 151 were completed. Responses to each question were measured using a Likert scale, ranging from 1 to 5 for the productivity and competitiveness questionnaires and from 1 to 3 for the corporate governance questionnaire. After 30 participants completed the questionnaires, Cronbach’s alpha was calculated, as reported above. Although data were collected from individual respondents, the company was treated as the unit of analysis. Multiple respondents from each company contributed domain-specific information. Responses were aggregated at the firm level by calculating the mean score for each questionnaire domain, thereby reducing individual-level bias and minimizing intra-firm dependence.

### 3.4. Statistical Analysis

Cronbach’s alpha was used to assess internal consistency reliability. Content validity was established through expert panel review. Descriptive statistics were used to summarize the observations. Scores and quantitative variables are reported as means, standard deviations, and 95% confidence intervals. Percentages are used to report qualitative variables. For statistical inference, the normality of questionnaire scores was first assessed using a Kolmogorov-Smirnov (KS) test, supplemented by skewness and kurtosis values; all values were within acceptable ranges, supporting the assumption of approximate normality (P = 0.517, 0.598, 0.136 for the KS test of normality, skewness = 0.015, -0.278, 0.156, and kurtosis = -0.869, 0.382, -1.037 for productivity, competitiveness, and corporate governance, respectively). Given the normal distribution of the scores, parametric tests were applied.

All inferential analyses were conducted at the firm level (productivity and competitiveness: n = 44; corporate governance: n = 43). Effect sizes, including Pearson correlation coefficients and mean differences with 95% confidence intervals, are reported alongside P values. Independent t-tests were used to assess the effects of binary variables (e.g., listed vs. non-listed, public vs. private, high-tech vs. non-high-tech production) on questionnaire scores. The choice between Welch’s t-test and Student’s t-test was guided by Levene’s test for homogeneity of variances.

Correlational analyses were conducted to examine associations among productivity, competitiveness, and corporate governance. Partial correlations were calculated to account for the influence of one variable on the association between the remaining two. Standardized effect sizes are reported where appropriate.

Mediation analysis was conducted using a bootstrapping approach with 2,000 resamples, and bias-corrected 95% confidence intervals were calculated for the average causal mediation effect (ACME), average direct effect (ADE), total effect, and proportion mediated. Both unstandardized and standardized estimates were reported. Given the cross-sectional design, mediation results are interpreted as associative rather than causal. Considering the sample size and exploratory nature of the study, analyses were primarily correlational. While regression-based modeling could further strengthen causal inference, the current analytical approach was intended to evaluate associations among variables and provide preliminary evidence for the proposed conceptual framework.

All analyses were performed using SPSS 26 and R (mediation package).

## 4. Results

One hundred seventy-six questionnaires were distributed to 44 companies, and 151 individual responses were received. Because the company was defined as the unit of analysis, responses from individuals within the same company were aggregated to generate firm-level scores for productivity, competitiveness, and corporate governance. Among the respondents, 121 (80.1%) were male and 30 (19.9%) were female. The number of respondents in each position is presented in Table 2. The mean productivity questionnaire score was 4.00 points (95% CI = 3.89 - 4.11, SD = 0.34) out of 5 on the Likert scale. The mean competitiveness score was 3.74 points (95% CI = 3.61 - 3.87, SD = 0.41) out of 5. The mean corporate governance questionnaire score was 2.57 points (95% CI = 2.50 - 2.63, SD = 0.20) out of 3. The distributions of productivity, competitiveness, and corporate governance scores are presented in Figures 1–3.

**Table 2.** Number of Respondents in Each Position

Position	Number of Participants	Number of Questionnaires Filled	Number of Questionnaires Left Unanswered	Response Rate (%)
**Member of the Board**	44	35	9	79.5
**CEO**	44	42	2	95.5
**CFO**	44	35	9	79.5
**CCO**	44	39	5	88.6

**Figure 1.**
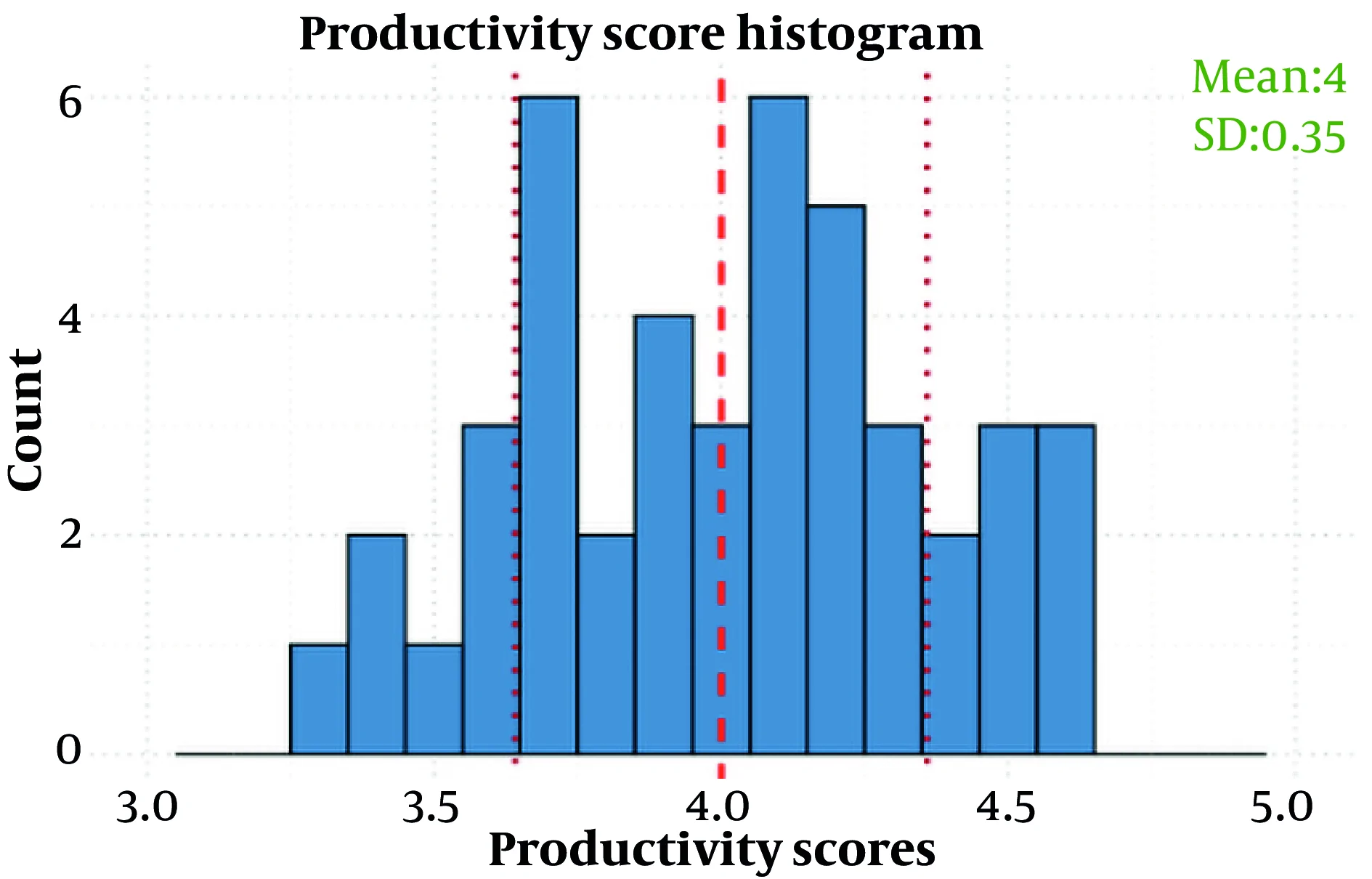
Histogram of productivity scores among respondents

**Figure 2.**
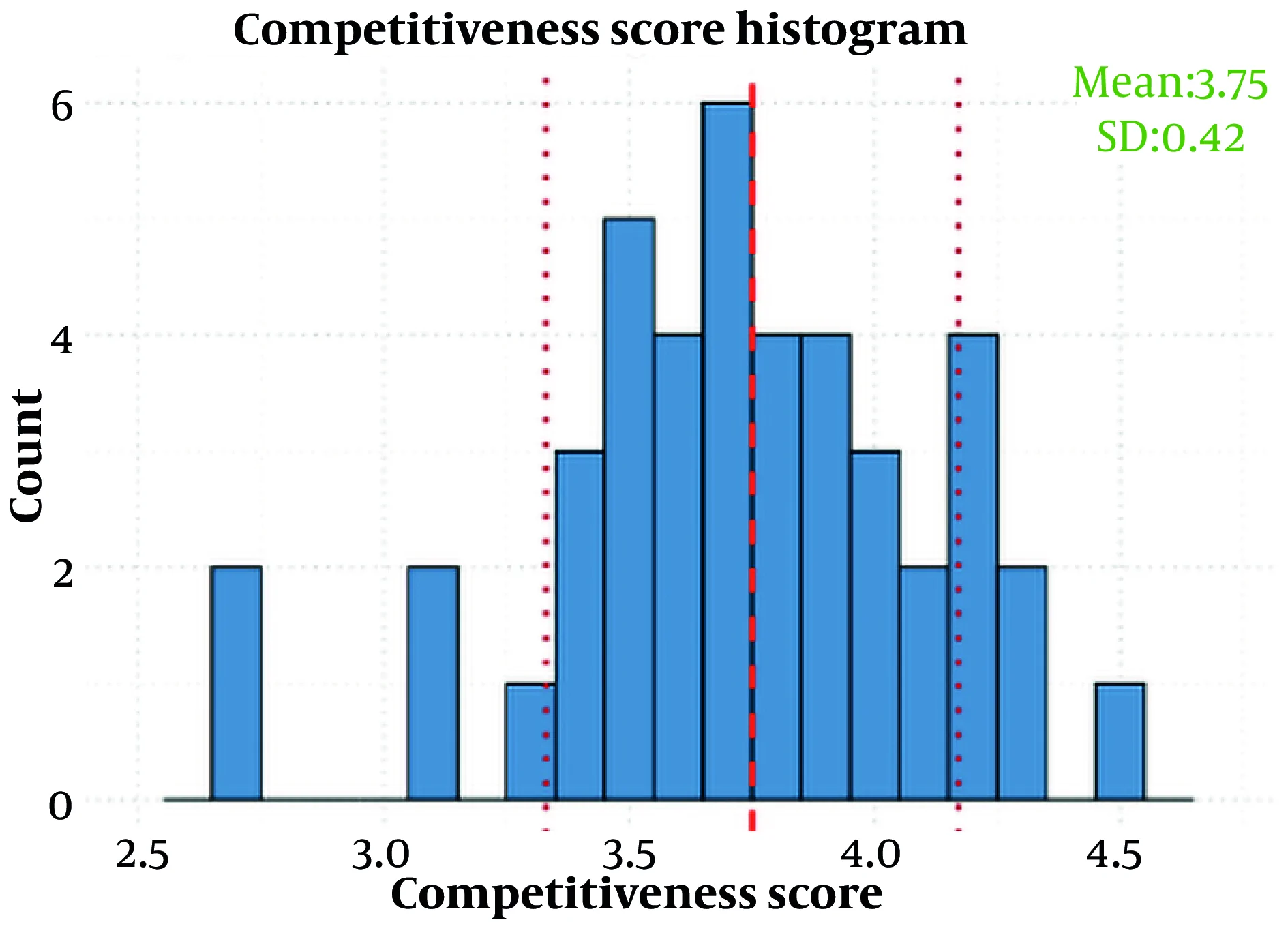
Histogram of competitiveness scores among respondents

**Figure 3.**
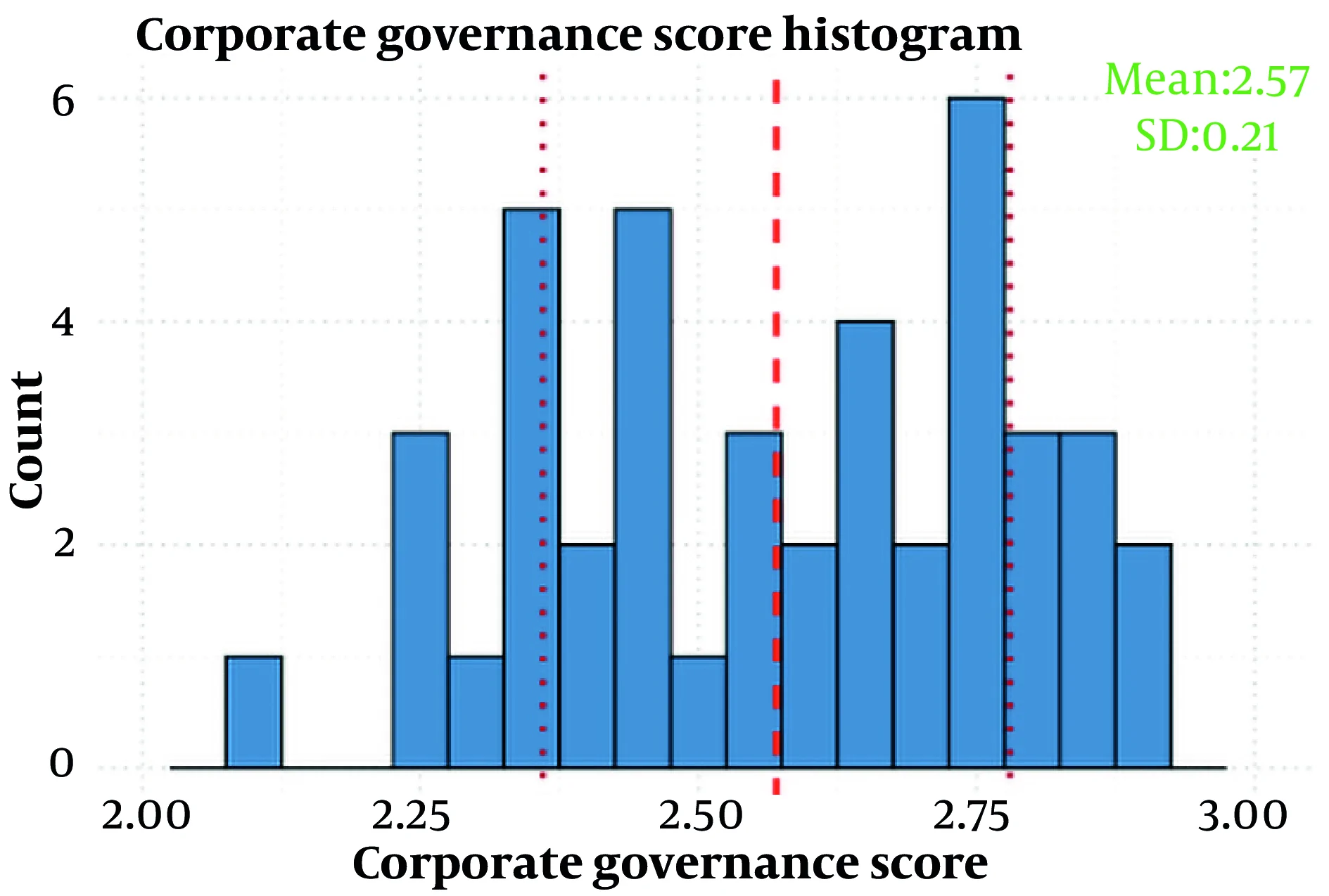
Histogram of corporate governance scores among respondents

### 4.1. Associations Between Secondary Company Characteristics and Primary Variables

The associations between questionnaire scores and binary company characteristics are summarized in Table 3.

**Table 3.** Association of Test Scores with Binary Independent Variables

Variables, Questionnaire and Group Status	Number	Mean	Standard Deviation	Mean Difference	P-value
**Is the company listed on the stock market?**					
Productivity				0.00716	0.959
No	36	4.0108	0.36165		
Yes	8	4.0036	0.29490		
Competitiveness				0.06522	0.692
No	36	3.7584	0.39487		
Yes	8	3.6932	0.51897		
Corporate governance				0.24658	0.000
No	36	2.5310	0.20140		
Yes	8	2.7776	0.07037		
**Is the company private or public?**					
Productivity				0.07007	0.612
Private	36	4.0222	0.34727		
Public	8	3.9522	0.36527		
Competitiveness				0.22649	0.164
Private	36	3.7878	0.36045		
Public	8	3.5613	0.59610		
Corporate governance				0.18368	0.030
Private	36	2.5413	0.20048		
Public	8	2.7249	0.18316		
**Is company a subsidiary of a holding company?**					
Productivity				0.04202	0.713
Yes	28	3.9853	0.32121		
No	14	4.0273	0.39525		
Competitiveness				0.10838	0.437
Yes	28	3.7815	0.43565		
No	14	3.6731	0.39095		
Corporate governance				0.11055	0.097
Yes	28	2.6057	0.19351		
No	14	2.4952	0.20569		
**Does the company possess its own distribution division?**					
Productivity				0.03668	0.735
Yes	23	4.0159	0.30933		
No	19	3.9792	0.38830		
Competitiveness				0.11476	0.384
Yes	23	3.7973	0.45762		
No	19	3.6825	0.37093		
Corporate governance				0.11433	0.071
Yes	23	2.6210	0.19879		
No	19	2.5066	0.19339		
**Is the company synthesizing High-tech medicines?**					
Productivity				0.12734	0.263
Yes	23	4.0433	0.35648		
No	15	3.9159	0.30577		
Competitiveness				0.32441	0.016
Yes	23	3.8545	0.41285		
No	15	3.5301	0.33566		
Corporate governance				0.00319	0.963
Yes	23	2.5652	0.22471		
No	15	2.5621	0.17937		
**Is the company producing biological medicines?**					
Productivity				0.30174	0.009
Yes	12	4.2014	0.29400		
No	24	3.8996	0.31784		
Competitiveness				0.38952	0.003
Yes	12	4.0176	0.31844		
No	24	3.6281	0.35582		
Corporate governance				0.11189	0.122
Yes	12	2.6261	0.17308		
No	24	2.5142	0.21072		
**Does the company have a history of export?**					
Productivity				0.20467	0.069
Yes	24	4.0931	0.28835		
No	15	3.8885	0.39404		
Competitiveness				0.14961	0.263
Yes	24	3.8307	0.35270		
No	15	3.6811	0.46798		
Corporate governance				0.11196	0.097
Yes	24	2.6000	0.20952		
No	15	2.4881	0.17916		

#### 4.1.1. Stock Market Listing

Among the 43 companies with corporate governance data, 7 were listed and 36 were not. The mean productivity and competitiveness scores for listed companies were 4.003 (SD = 0.294, SE = 0.104) and 3.693 (SD = 0.518, SE = 0.183), respectively, compared with 4.010 (SD = 0.361, SE = 0.060) and 3.758 (SD = 0.394, SE = 0.065) for non-listed companies. Corporate governance scores were 2.776 (SD = 0.070, SE = 0.026) for listed companies and 2.531 (SD = 0.201, SE = 0.033) for non-listed companies. The difference in corporate governance between listed and non-listed companies was statistically significant (mean difference = 0.245, 95% CI = 0.158 - 0.332, P < 0.001), whereas differences in productivity and competitiveness were not significant (P = 0.959 and 0.692, respectively).

#### 4.1.2. Public vs. Private Companies

Among the 43 companies with corporate governance data, 7 were public and 36 were private. The mean productivity, competitiveness, and corporate governance scores for public companies were 3.952 (SD = 0.365, SE = 0.129), 3.561 (SD = 0.596, SE = 0.210), and 2.724 (SD = 0.183, SE = 0.069), respectively. Private companies had scores of 4.022 (SD = 0.347, SE = 0.057), 3.787 (SD = 0.360, SE = 0.060), and 2.541 (SD = 0.200, SE = 0.033), respectively. The difference in corporate governance was statistically significant (mean difference = -0.183, 95% CI = -0.348 to -0.018, P = 0.030), whereas differences in productivity and competitiveness were not.

#### 4.1.3. High-tech Medicine Production

Among 38 companies, 23 produced high-tech medicines and 15 did not. High-tech producers scored 4.043 (SD = 0.356, SE = 0.074) for productivity, 3.854 (SD = 0.412, SE = 0.086) for competitiveness, and 2.565 (SD = 0.224, SE = 0.046) for corporate governance. Non-producers scored 3.915 (SD = 0.305, SE = 0.078), 3.530 (SD = 0.335, SE = 0.086), and 2.562 (SD = 0.179, SE = 0.046), respectively. The difference in competitiveness was statistically significant (mean difference = 0.324, 95% CI = 0.065 - 0.583, P = 0.016), whereas differences in productivity and corporate governance were not.

#### 4.1.4. Biological Medicine Production

Among 36 companies, 12 produced biological medicines and 24 did not. Producers scored 4.201 (SD = 0.294, SE = 0.084) for productivity, 4.017 (SD = 0.318, SE = 0.091) for competitiveness, and 2.626 (SD = 0.173, SE = 0.049) for corporate governance. Non-producers scored 3.899 (SD = 0.317, SE = 0.064), 3.628 (SD = 0.355, SE = 0.072), and 2.514 (SD = 0.210, SE = 0.043), respectively. Differences in productivity and competitiveness were statistically significant (P = 0.009 and 0.003, respectively), whereas the difference in corporate governance was not.

Other company characteristics (subsidiary status, distribution division, exporting history, employment of family members) were not meaningfully associated with productivity, competitiveness, or corporate governance scores (P values > 0.05).

### 4.2. Correlations Among Productivity, Competitiveness, and Corporate Governance

Zero-order Pearson correlations indicated no statistically significant correlation between competitiveness and corporate governance (r = 0.299, P = 0.052, n = 43), although the coefficient suggests a weak association that should be interpreted cautiously. Productivity was strongly correlated with competitiveness (r = 0.780, P = 0.001, n = 44) and moderately correlated with corporate governance (r = 0.419, P = 0.005, n = 43).

The partial correlation, with productivity as the control variable, yielded a correlation coefficient of -0.056 (P value = 0.726) for the relationship between competitiveness and corporate governance. When corporate governance was used as the control variable, productivity was correlated with competitiveness (Pearson r = 0.764, P < 0.001). Similarly, when competitiveness was used as the control variable, productivity was correlated with corporate governance (Pearson r = 0.312, P value = 0.044) (Figure 4).

**Figure 4.**
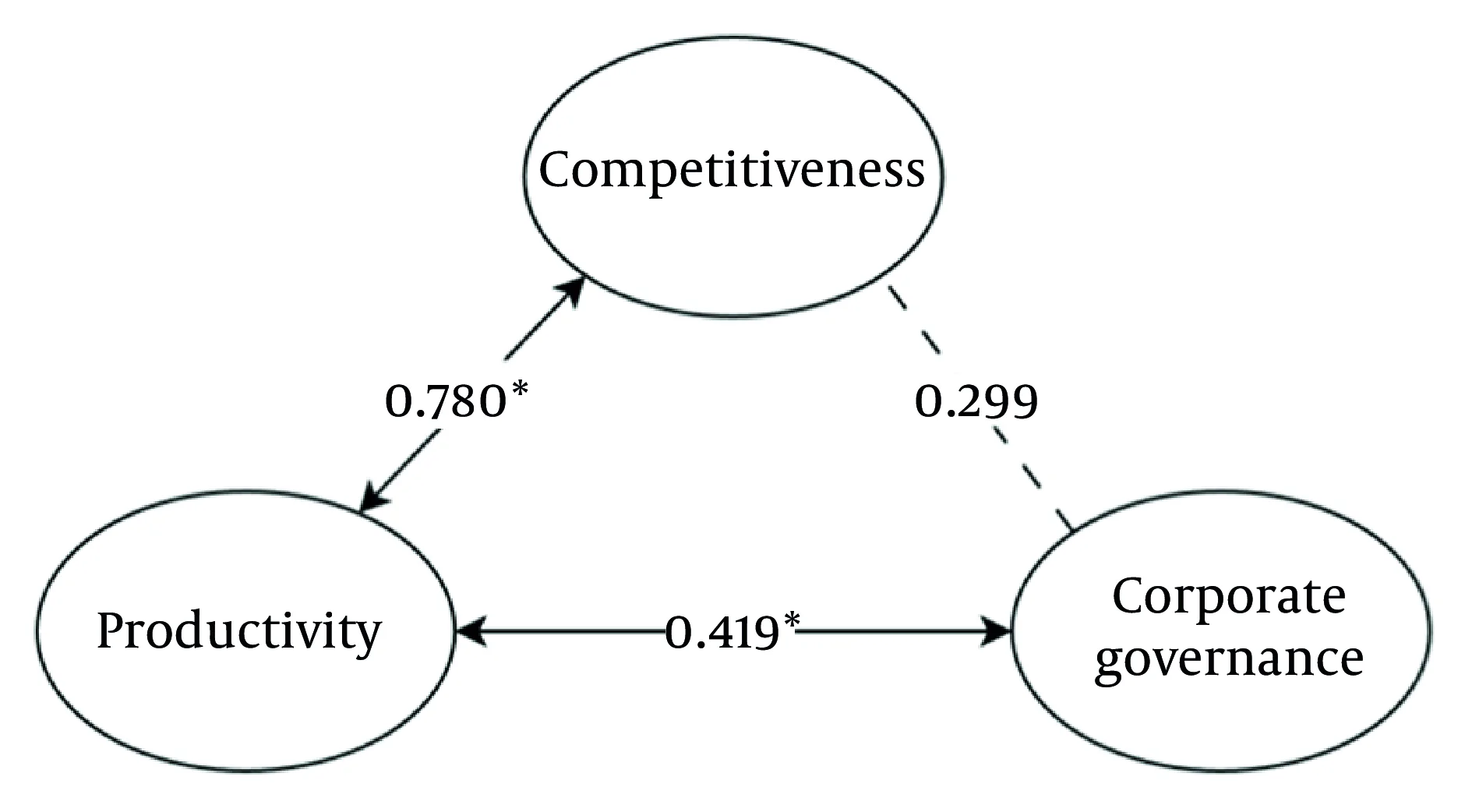
Model with the corresponding Pearson coefficients

### 4.3. Mediation Analysis with Bootstrapping

The bootstrapping assessment of the association between corporate governance and competitiveness, with productivity as a mediator, was conducted in 43 companies. The mediation analysis used 2,000 bootstrap simulations with bias-corrected 95% confidence intervals. The unstandardized results were: ACME = 0.679 (95% CI = 0.218 - 1.150, P = 0.004), ADE = -0.076 (P = 0.770), total effect = 0.603 (95% CI = 0.009 - 1.130, P = 0.048), and proportion mediated = 1.126 (95% CI = 0.330 - 3.704, P = 0.044).

The corresponding standardized effects were: standardized ACME = 0.312, standardized ADE = -0.035, standardized total effect = 0.277, and standardized proportion mediated = 1.126. Given the cross-sectional design, these results should be interpreted as associations rather than causal effects.

## 5. Discussion

This study aimed to assess the relationships among corporate governance, productivity, and competitiveness in Iranian pharmaceutical companies and to evaluate how these aspects may influence one another. Our initial theoretical framework hypothesized a fully interdependent relationship among corporate governance, productivity, and competitiveness, suggesting significant zero-order correlations across all three pairwise combinations. Specifically, we anticipated a direct association between corporate governance and competitiveness, as well as their respective correlations with productivity. However, the findings of the current study deviated from this expected model. Although productivity was significantly correlated with both corporate governance and competitiveness, our analysis revealed no direct association between corporate governance and competitiveness. This lack of a direct correlation persisted after controlling for the influence of productivity, as evidenced by a non-significant partial correlation coefficient between these two variables. The bootstrapping results further supported this interpretation. The confidence interval of ACME, as an indicator of the indirect effect between corporate governance and competitiveness, was above 0, indicating a positive indirect relationship between these aspects. However, the confidence interval of ADE contained zero, suggesting that even if a direct relationship between corporate governance and competitiveness exists, it is likely weak. Moreover, the total effect of corporate governance on competitiveness was positive and borderline significant, which was consistent with the correlation analysis. Additionally, the estimated proportion mediated of 1.12 indicates a substantially greater contribution of the indirect effect to the total effect than the direct effect. Overall, these findings suggest a strong mediating role for productivity. However, because this study was cross-sectional, the observed relationships should be interpreted as associations rather than causal effects. Although mediation analysis can provide insight into potential structural relationships among variables, the absence of temporal ordering limits causal inference.

This outcome suggests a partially interdependent model in which corporate governance and competitiveness are not directly linked, and their relationship may be mediated by productivity. Our results suggest that corporate governance contributes to competitiveness primarily through its positive impact on productivity. Although no direct causal interpretation is implied, the findings can be described as follows: effective corporate governance practices foster higher productivity, which, in turn, enhances a firm’s competitiveness in the market. This revised understanding proposes an indirect relationship between corporate governance and competitiveness, with productivity serving as a key intervening variable. This contrasts with models that posit a direct, unmediated link between corporate governance and market-level competitiveness.

Consistent with numerous studies, a strong positive relationship was found between productivity and competitiveness (5, 6, 29-33). Higher productivity reduces costs, improves quality, and accelerates innovation, all of which enhance a company’s competitive capacity. At the same time, greater competitiveness may help preserve market share, reduce marketing costs, enable companies to focus on internal processes, and create opportunities for investment in productivity. Similarly, a significant positive relationship was observed between productivity and corporate governance, aligning with findings from Tian et al. and Gao et al., which demonstrate that exemplary and structured implementation of corporate governance practices contributes to higher productivity through better management decisions, efficient resource utilization, and reduced operational risks (28, 34). These mechanisms may suggest a causal component; however, given the cross-sectional design, causality cannot be established. Further studies using designs suited to causal inference are needed.

In contrast to our initial expectations, we found no significant direct relationship between corporate governance and competitiveness. This finding diverges from those of Ho et al. and Bhatt et al. in the Chinese and Malaysian economic ecosystems, which reported positive effects of corporate governance on performance and competitiveness (24, 35). However, our findings align with observations from Saudi Arabia by Fallatah (36). Although corporate governance enhances internal processes and productivity, its direct impact on market dynamics and competitiveness appears limited. Competitiveness is often influenced by a broader array of external factors, such as market conditions, customer preferences, and technological advancements, which may overshadow the direct contribution of internal governance. Furthermore, the benefits of improved corporate governance for competitiveness may manifest indirectly or over a longer period, with productivity gains requiring time to translate into competitive advantages. This highlights the complex and multifaceted nature of these interactions and underscores the importance of considering the broader environmental context.

Among the independent variables, four were attributed to the company’s structure and organizational chart: type of ownership, being a subsidiary of a holding company, being listed on the stock market, and having an internal distribution division. Our findings suggest that both publicly owned companies and those listed on the stock market were positively correlated with the corporate governance score; no other significant relationships were found between these variables and the scores. The positive correlation between being publicly traded and corporate governance was expected, as companies are required to follow specific guidelines, particularly regarding their corporate governance structure, to undergo an initial public offering (IPO). However, the higher corporate governance score among non-private companies was unexpected, as these companies are strongly influenced by governmental organizations, and senior management positions are usually assigned by these organizations. This would theoretically lead to a lack of meritocracy, which in turn reduces motivation and independence among managers, resulting in weaker implementation of corporate governance and poorer company performance. Our findings contradict this causal assumption and suggest an alternative explanation: at least in the context of privately owned pharmaceutical companies, owners may be overprotecting their investments through direct control over operations.

The two product-related variables were production of biologic products and production of high-tech medicines, both of which were correlated with a higher competitiveness score. This was expected, as numerous studies confirm that the development of differentiated products, innovation, and design can significantly enhance competitiveness and productivity (8, 24, 26, 37, 38). Notably, we found no significant difference in the productivity of high-tech producers, whereas productivity increased among biologic producers. This finding, which contradicts existing evidence, may be explained by the inherent financial risks associated with producing high-tech medicines. This added risk in the already high-risk environment of Iran’s economic ecosystem may be associated with lower productivity. In the case of biologic medicines, the cumulative experience of Iranian pharmaceutical companies in synthesizing biological medicines has helped mitigate financial risks, thereby enhancing productivity in companies that synthesize biological products.

The findings may inform efforts to improve competitiveness and productivity through stronger implementation of corporate governance by:

1) conveying the importance of corporate governance to senior decision-makers and explaining why practical governance frameworks should replace purely formal approaches;

2) selecting senior decision-makers, such as board members and chief executive officers, based on expertise and experience through a third-party scoring system to minimize personal judgment in appointments;

3) using financial and nonfinancial compensation systems to motivate managers and improve productivity; and

4) adopting strategies focused on optimizing productivity and competitiveness by identifying barriers to improvement and using key performance indicators to measure changes validly and promptly.

### 5.1. Study Limitations and Future Research

Despite efforts to explore the interrelationships among corporate governance, competitiveness, and productivity, the study encountered limitations, primarily related to statistical power. Our initial theoretical framework posited a fully interdependent relationship among these three variables. However, the sample size of 43 companies may have been insufficient to robustly detect all hypothesized associations. For instance, the zero-order correlation between corporate governance and competitiveness, although moderate in magnitude (r = 0.299), did not reach statistical significance (P = 0.052). A post hoc power analysis revealed that the study had only a 50.4% chance of detecting a true correlation of this magnitude, suggesting a substantial risk of a Type II error. This limitation became even more pronounced when examining the unique relationship between corporate governance and competitiveness, after controlling for productivity, using partial correlation. The observed partial correlation was very weak (r = -0.056), and the power to detect such an effect was extremely low (approximately 6.3%). Consequently, the non-significant findings, particularly regarding the direct relationship between corporate governance and competitiveness, should be interpreted with caution, as the study lacked sufficient statistical power to reliably confirm the absence of these associations. It is important to note that this low statistical power is a direct consequence of the weak effect sizes observed in our sample. Thus, while our study may have been underpowered to detect a significant correlation, the data suggest that even if a true correlation exists in the population, it is likely to be weak in magnitude.

Although the validity and internal consistency of the questionnaires were optimal, it should be noted that performance measures, particularly productivity, are more accurately assessed using direct company output-input data. However, technical limitations, primarily the confidentiality of these data, prevented the use of such measures.

Studies comparing the status of productivity, competitiveness, and corporate governance among Iranian companies and those in other countries are also necessary to objectively assess the current state of these aspects in the Iranian pharmaceutical industry. A more in-depth assessment of financial risks and their association with these aspects could improve understanding of these metrics, as financial risks are among the biggest concerns in Iran’s industrial ecosystem. Additionally, more objective measurement of productivity could improve the precision and power of research, and future studies could better explain these relationships through direct measurement of company productivity rather than using a questionnaire. Moreover, future studies focusing on assessing causal relationships are encouraged.

The main limitations of this study arose from practical constraints in data measurement. Using questionnaires to measure company-level variables can introduce substantial bias because these variables are not directly measured. Moreover, the data were collected using a cross-sectional design. Given that governance factors are highly complex and evolve over time, future research should use dynamic panel data to trace how changes in governance structures affect TFP over time. This could lead to a better understanding of the causal chain and a more accurate representation of interactions among the study dimensions. Additionally, while 44 companies were used for modeling, qualitative deep dives into the correlations of these outputs within a single company were not conducted. We encourage future studies to address this gap.

### 5.2. Conclusions

The findings of this study support an associational model in which corporate governance is linked to competitiveness primarily through its relationship with productivity, conceptualized as a multidimensional proxy for Total Factor Productivity. Companies with stronger governance structures tended to demonstrate higher productivity scores, and higher productivity was strongly associated with improved competitiveness. However, no statistically significant direct association between corporate governance and competitiveness was observed. Given the cross-sectional design, these relationships should be interpreted as structural associations rather than confirmed causal pathways. The results suggest that strengthening governance structures may correspond to improved organizational efficiency and resource utilization, which in turn may be linked to enhanced competitive positioning. Longitudinal and data-driven productivity studies are required to confirm the temporal and causal mechanisms underlying these relationships.

## Data Availability

The dataset presented in the study is available on request from the corresponding author during submission or after publication. The data are not publicly available due to the sensitive nature of the company's performance.
